# A Web-Based Photo-Alteration Intervention to Promote Sleep: Randomized Controlled Trial

**DOI:** 10.2196/12500

**Published:** 2019-09-26

**Authors:** Isabel Perucho, Kamalakannan M Vijayakumar, Sean N Talamas, Michael Wei-Liang Chee, David I Perrett, Jean C J Liu

**Affiliations:** 1 Division of Social Sciences Yale-NUS College Singapore Singapore; 2 School of Psychology University of Pennsylvania Philadelphia, PA United States; 3 Neuroscience and Behavioral Disorders Programme Duke-NUS Medical School Singapore Singapore; 4 School of Psychology and Neuroscience University of St Andrews St Andrews United Kingdom

**Keywords:** sleep, public health, physical appearance, outward appearance

## Abstract

**Background:**

Receiving insufficient sleep has wide-ranging consequences for health and well-being. Although educational programs have been developed to promote sleep, these have had limited success in extending sleep duration. To address this gap, we developed a Web-based program emphasizing how physical appearances change with varying amounts of sleep.

**Objective:**

The aims of this study were to evaluate (1) whether participants can detect changes in appearances as a function of sleep and (2) whether this intervention can alter habitual sleep patterns.

**Methods:**

We conducted a 5-week, parallel-group, randomized controlled trial among 70 habitual short sleepers (healthy adults who reported having <7 hours of sleep routinely). Upon study enrollment, participants were randomly assigned (1:1) to receive either standard information or an appearance-based intervention. Both groups received educational materials about sleep, but those in the appearance group also viewed a website containing digitally edited photographs that showed how they would look with varying amounts of sleep. As the outcome variables, sleep duration was monitored objectively via actigraphy (at baseline and at postintervention weeks 1 and 4), and participants completed a measure of sleep hygiene (at baseline and at postintervention weeks 2, 4, and 5). For each outcome, we ran intention-to-treat analyses using linear mixed-effects models.

**Results:**

In total, 35 participants were assigned to each group. Validating the intervention, participants in the appearance group (1) were able to identify what they looked like at baseline and (2) judged that they would look more attractive with a longer sleep duration (*t*_26_=10.35, *P*<.001). In turn, this translated to changes in sleep hygiene. Whereas participants in the appearance group showed improvements following the intervention (*F*_1,107.99_=9.05, *P*=.003), those in the information group did not (*F*_1,84.7_=0.19, *P*=.66). Finally, there was no significant effect of group nor interaction of group and time on actigraphy-measured sleep duration (smallest *P*=.26).

**Conclusions:**

Our findings suggest that an appearance-based intervention, while not sufficient as a stand-alone, could have an adjunctive role in sleep promotion.

**Trial Registration:**

ClinicalTrials.gov NCT02491138; https://clinicaltrials.gov/ct2/show/study/NCT02491138.

## Introduction

### Background

Sleep is often described as 1 of 3 pillars of health, ranked alongside nutrition and exercise as modifiable targets of well-being. Underscoring this point, a large body of evidence suggests that the habitual curtailment of sleep increases the risk of obesity [1-3], coronary heart disease [4], stroke [4], and all-cause mortality [5,6]. Despite the centrality of sleep, 1 in 3 adults routinely obtain less than the recommended 7 hours of sleep for healthy adults [7,8]. Accordingly, the US Department of Health and Human Services has outlined a nation-wide goal to reduce the number of habitual short sleepers over a 10-year period [9].

Despite this goal, sleep promotion campaigns have met with limited success. In a typical program, participants are briefed about the mechanics of sleep (eg, sleep architecture), the importance of sleep, and sleep hygiene—lifestyle habits that facilitate sleep (eg, abstaining from excessive caffeine intake during the night [10]) [11-13]. Delivery of these programs range from simply providing a pamphlet [14] to hosting multiple sessions (eg, 8 sessions over 5 weeks [15]). Although sleep promotion programs may raise awareness, they fare poorly in changing actual behavior [11,12]. Even among programs that have reported increased sleep duration, gains have been short-lived, observed only immediately after the intervention [13].

Reviewing this evidence, Cassoff et al suggested that merely providing information may not be sufficient and that greater emphasis should be placed on motivational aspects of modifying sleep [12]. This concurs with surveys suggesting that—despite knowing the importance of sleep—many forsake it for immediate priorities such as meeting deadlines [16,17]. That is, they engage in *temporal discounting*, weighting short-term rewards (eg, completing one’s assignment) over long-term outcomes (eg, reduced risk of all-cause mortality) [18,19]. This aligns with research in other health domains (eg, appetite regulation), where interventions emphasizing short-term gains (eg, financial incentives for weight loss or exercise) were found to encourage healthy behaviors [20,21]. Correspondingly, one way to motivate better sleep patterns may be to highlight short-term outcomes [22,23]—the proximate incentives of increased sleep duration or the proximate costs of sleep curtailment. To this end, we evaluated a sleep promotion program emphasizing 1 immediate outcome—how physical appearances change as a function of sleep duration.

### Developing an Appearance-Based Intervention for Sleep Promotion

Physical appearance is highly valued across cultures [24], and the desire to be attractive has motivated healthy behaviors in both men and women [25]. For example, in the prevention of skin cancers, showing participants how they would look with continued sun exposure has been found repeatedly to increase sun-protection behaviors and reduce indoor tanning frequency [25]. In smoking cessation campaigns, showing the photoaging effects of tobacco has likewise increased readiness to change and actual quit attempts [26,27]. Finally, a recent study found that when participants were shown how their skin color would vary as a function of nutrition, their intake of fruit and vegetable consumption increased, with effects lasting for 10 weeks postintervention [28].

In the context of sleep, physical appearances track sleep duration within a short time window (eg, after 31 hours of total sleep deprivation [29,30] or following 2 nights with 4 hours of sleep opportunity [31]). Relative to adequate sleep (≥7 hours), sleep loss results in hanging eyelids, redder and swollen eyes, darker eye circles, wrinkles and fine lines around the eyes, a droopy mouth, and poorer skin quality (eg, worsened skin hydration and elasticity and paler skin) [30,32]. In turn, these alterations are associated with looking sadder [30], less intelligent [33], less attractive [29], less healthy [29], and less desirable as a social companion [31]. Given the immediate and salient nature of these changes, physical appearances could serve as a motivator to overcome temporal discounting. Accordingly, we developed a novel Web-based intervention to capitalize on these changes.

To summarize, we sought in this study to highlight the immediate impact of sleep loss on physical appearances. Drawing from other health domains (sun protection, smoking cessation, and diet), we hypothesized that the appearance-based intervention would augment a standard informational campaign in increasing sleep duration. Furthermore, because poor sleep hygiene predisposes a person to impaired nocturnal sleep and is targeted in sleep education programs [10,11], we tracked changes in sleep hygiene habits as an additional indicator of behavioral change [34].

## Methods

### Study Design

This study involved a randomized controlled open-label trial with 2 arms: a standard information intervention and an appearance-based intervention. The trial design and outcomes were preregistered in ClinicalTrials.gov (NCT02491138) and remained unchanged throughout the study. All procedures were approved by the National University of Singapore’s Institutional Review Board (A-15-083).

### Participants

A total of 70 young adults were recruited from the National University of Singapore between July 2015 and December 2016. Participants responded to advertisements within the university and registered their interest via an online website. Thereafter, they were included if they were (1) aged between 18 and 24 years; (2) had no history of psychiatric, sleep, neurological, or medical disorders (including insomnia); (3) had no history of substance abuse; and (4) reported habitual short sleep (as defined by current sleep guidelines: sleep duration of <7 hours [8]).

Following written informed consent (see Multimedia Appendix 1), participants were randomized to the 2 groups with a 1:1 allocation. The allocation sequence was prepared by the study coordinator before trial commencement and involved a computer-generated list of random numbers. Both participants and research assistants became aware of the allocation on the intervention day; however, research staff involved in data entry and data cleaning were blinded to participant grouping. Participants were not told what the intervention of interest was.

### Procedures

As a baseline measure, participants completed 1 week of sleep monitoring where they kept a sleep diary [35] and wore a wrist actigraph on the nondominant hand (Actiwatch; Philips Respironics, Inc, Pittsburgh, PA). Activity was recorded in epochs of 2 min, and actigraphy measures were calculated using Actiware 6.0.5 (Philips Respironics, Inc, Pittsburgh, PA) after photosensor data and sleep diaries were reviewed.

During the baseline phase, participants also attended the laboratory for a face-to-face visit where they completed the Sleep Hygiene scale [36]. This scale involved 19 items assessing the number of days each week participants engaged in poor sleep hygiene practices (eg, *slept in a room that was too bright*; *worried, planned, or thought about important matters in bed*). The 19 items were then averaged to form a composite score, with higher scores indicating poorer sleep hygiene (Cronbach α=.72). As this component is targeted in typical sleep education programs, any improvements from baseline would represent a preliminary step in behavioral change.

As additional baseline measures, participants were also characterized with 2 questionnaires assessing sleep quality and attitudes: (1) the Dysfunctional Beliefs and Attitudes about Sleep scale (DBAS-16) [37], a 16-item measure of insomnia-related cognition (Cronbach α=.87); and (2) the Pittsburgh Sleep Quality Index (PSQI) [38], a 19-item measure of sleep quality over the past week (Cronbach α=.52). Finally, facial photographs were obtained from all participants (regardless of their intervention group).

For the photoshoot, 3 facial photographs were taken in a standardized room under constant lighting (resolution: 3648 × 5472 pixels, Canon Powershot G7X; Canon Inc, Tokyo, Japan). Before taking photographs, participants removed any spectacles and jewelry, combed their hair backwards, were clean shaven, and applied a facial wipe. They were then instructed to look into the camera with a neutral expression, with photographs taken at a fixed distance from the camera.

**Figure 1:**
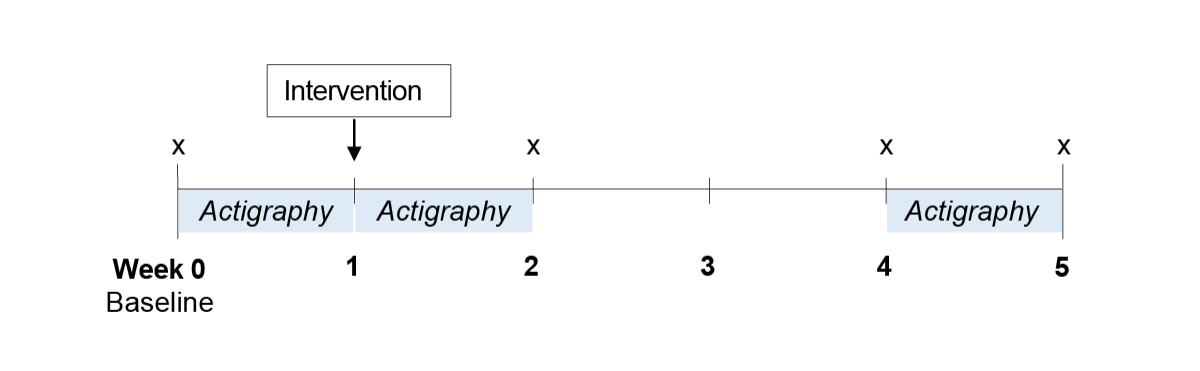
Schematic of study procedures. All participants received the intervention in week 1 and had their sleep patterns monitored through actigraphy in weeks 0 (baseline), 1, and 4. The sleep hygiene questionnaire was also administered in weeks 0 (baseline), 2, 4, and 5 (marked in the figure with an “x”).

After the baseline phase (week 0), participants visited the laboratory individually where they received either the information or appearance-based intervention. Following this visit, participants repeated the sleep hygiene scale (weeks 2, 4, and 5) and completed 2 weeks of wrist actigraphy (weeks 1 and 4). Finally, they repeated the DBAS-16 and PSQI questionnaires (in weeks 2, 4, and 5). Upon study completion, participants were reimbursed SGD $50. All study visits took place at the behavioral laboratories of Yale-NUS College in Singapore, with procedures and measures administered in English—the lingua franca of the country (see Figure 1 for a schematic of study procedures).

### Interventions

#### Standard Information

In the standard information group, participants were shown a slideshow containing materials based on public health websites [39,40] and previous sleep education studies [11-13]. A trained research assistant walked participants through the slide contents, introducing them to: the functions of sleep, the consequences of sleep curtailment, behavioral signs of insufficient sleep, and sleep hygiene principles to promote sleep (eg, noise management in the sleeping environment). Participants were then given a take-home pamphlet containing this same information and were not given the opportunity to ask questions. Each session was conducted individually and spanned approximately 20 min.

#### Appearance-Based Intervention

**Figure 2:**
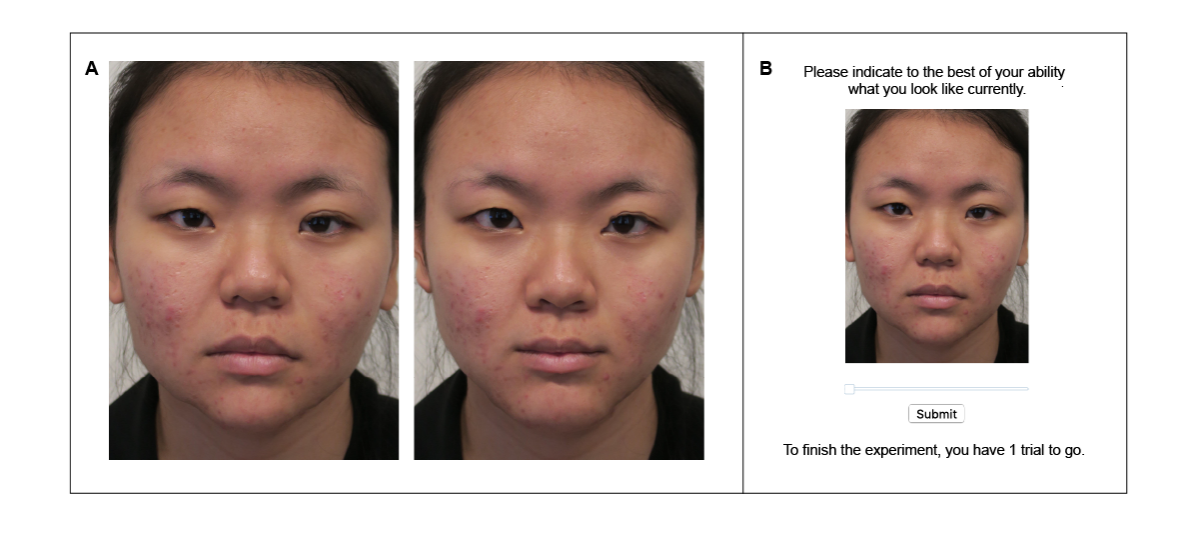
In the appearance-based intervention, participants were shown what they would look like as a function of varying sleep duration. (A) Each participant’s face was digitally edited to show them their sleep restricted (left image) and well-rested selves (right image). (B) A continuum of 13 images was created and presented to participants through an online website.

In the appearance group, transformed versions of baseline photographs were presented through an internet platform to show how appearances would change with sleep duration. Sleep-related changes were ascertained from an earlier study where 25 healthy adults were photographed following rested wakefulness (7-9 hours of sleep) and following 2 nights of sleep restriction (where they were given 4 hours of sleep opportunity) [31]. Using Psychomorph 6 [41] — a free software for photo transformation—these faces were blended to create 2 masks for each state (rested vs sleep restricted). Transformations were then applied by first delineating participants’ photographs with 175 feature points aligned based on interpupillary distance. Using the masks, participants’ photographs were transformed to create a continuum of 13 images, with Image 0 showing what participants would look like if they had insufficient sleep (300% of the difference between the rested and sleep restricted masks; Figure 2). This progressed incrementally with Image 6 as the original image, and Image 12 showing what participants would look like if they received more sleep (300% of the difference in the opposite direction; Figure 2).

During the intervention visit, participants were shown the same slideshow presentation as the information group. However, after hearing about the signs of insufficient sleep, they were shown a website where the 13 transformed images were presented on a continuum, with a sliding scale to manipulate appearance changes as a function of sleep duration (Figure 2). Participants scrolled through the website in a self-paced manner and were asked to (1) identify what they looked like at present and (2) to adjust the scale to select their most attractive self. Upon making their choices, participants were again presented with Images 0 (their sleep-restricted selves) and 12 (their well-rested selves). Referring to these images, the trained research assistant highlighted how sleep curtailment affected their appearances (eg, hanging eyelids and droopy mouth), and how that could be perceived by others (eg, as less attractive). Thereafter, participants were reminded of the costs many incur for physical attractiveness (eg, paying a gym membership for $100/month), relative to the benefits accrued from extending sleep duration. Finally, participants were presented information about sleep hygiene practices and were given a take-home pamphlet (similar to the information group). Each session was conducted individually and spanned approximately 30 min, with neither an invitation for participants to ask questions nor further access to the website (following the intervention).

### Statistical Analysis

For the primary outcome measures (actigraphy-measured sleep duration and sleep hygiene scores), we ran intention-to-treat analyses using linear mixed-effects models, with parameters estimated using maximum likelihood estimation for a first-order autoregressive covariance structure. This examined individual change in each measure over time, with group (information or appearance), time (sleep duration: weeks 0, 2, 4, and 5; sleep hygiene: 0, 1, and 4), and the group × time interaction entered as fixed effects. Random intercepts accounted for correlated data due to repeated measures. Although the reliability of the PSQI was low in our sample (Cronbach α=.52), baseline scores differed between the groups and were entered as covariates [42]; however, the conclusions did not change with and without this adjustment. Finally, for sleep duration, separate models were run for weekdays and for weekends to account for differences in sleep patterns across the week [43].

For the secondary outcome measures (global PSQI and DBAS-16 scores), we ran linear mixed-effects models identical to those used for the primary outcome measures, except that the model with global PSQI scores did not include covariates.

All analyses were conducted using SPSS 25 (IBM Corp, Armonk, NY) and R 3.4.0 (R Core Team, Vienna, Austria), with the type 1 decision-wise error rate controlled at α=.05. Power calculations for the main intention-to-treat analyses showed that there was statistical power at the recommended .80 level to detect a medium effect size (computed through simulations, based on a meta-analysis evaluating appearance-based interventions for sun protection behaviors [25]). Accordingly, data collection was scheduled to cease when 70 participants had been recruited.

## Results

### Participant Characterstics

**Figure 3:**
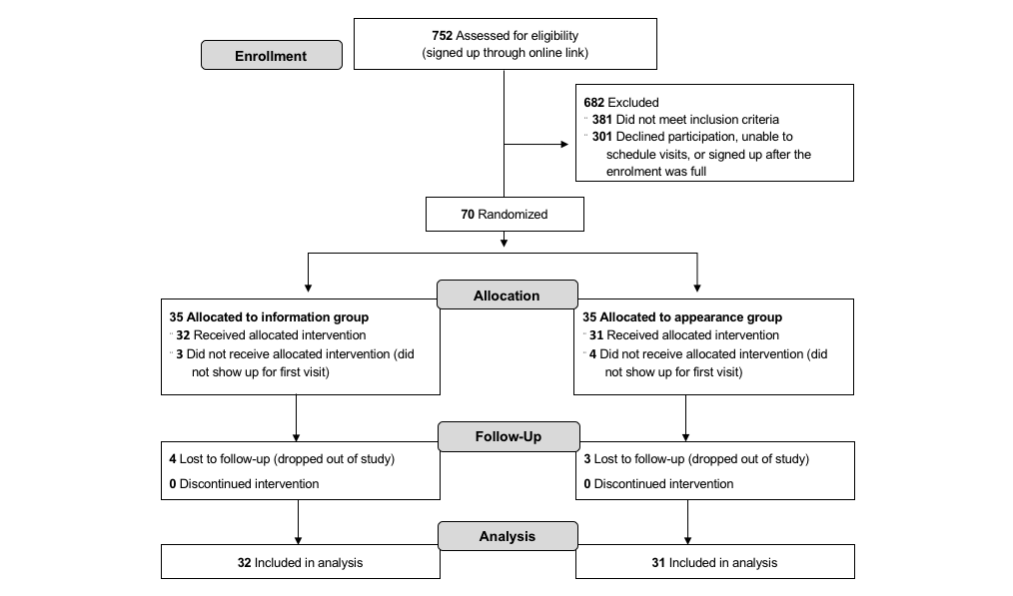
Consolidated Standards of Reporting Trials flow diagram.

Full details on participant flow are shown in Figure 3. At baseline, actigraphy monitoring showed that participants slept an average of 5.9 hours on weekdays (SD 0.90 hours) and 6.4 hours on weekends (SD 1.32 hours). There were no significant group differences in baseline: gender, ethnicity, age, DBAS, sleep hygiene scores, and actigraphy-measured sleep variables (Tables 1 and 2). However, baseline global PSQI differed between groups and was included as a covariate.

**Table 1:** Baseline characteristics of participants in the information and appearance groups.

Characteristics		Information (n=32), n (%)	Appearance (n=31), n (%)	Chi-square (*df)*	*P* value
**Gender**					
	Female	25 (78)	18 (58)	2.92 (1)	.09
**Ethnicity**				7.83 (8)	.45
	Chinese	23 (72)	22 (71)	—^a^	—
	Indian	4 (12)	7 (22)	—	—
	Others	5 (16)	2 (6)	—	—

^a^Not applicable.

**Table 2:** Baseline characteristics of participants by sleep score and actigraphy metrics.

Characteristics		Information (n=32), mean (SD)	Appearance (n=31), mean (SD)	*t* test	*P* value
Age (years)		20.66 (1.91)	22.13 (2.14)	−0.93 (61)	.36
**Mean sleep questionnaire scores**					
	Pittsburgh Sleep Quality Index (global score)	10.72 (2.70)	9.57 (1.65)	1.99 (57)	.05
	Dysfunctional Beliefs and Attitudes about Sleep	4.42 (1.32)	4.67 (1.44)	−0.71 (61)	.48
	Sleep Hygiene Score	2.61 (0.74)	2.52 (0.65)	0.51 (61)	.61
**Mean actigraphy metrics (averaged across the week)**					
	Bed time (hours:min)	2:18 (1:02)	1:57 (1:14)	1.19 (56)	.24
	Wake time (hours:min)	8:57 (1:05)	8:47 (1:12)	0.57 (56)	.57
	Weekday sleep duration (hours:min)	5:55 (0:52)	5:54 (0:56)	0.04 (56)	.97
	Weekend sleep duration (hours:min)	6:07 (1:18)	6:42 (1:18)	−1.71 (55)	.09
	Sleep latency (mins)	12.77 (8.01)	14.00 (8.81)	0.55 (56)	.58
	Wake after sleep onset (mins)	30.78 (17.24)	29.99 (17.36)	0.17 (56)	.86
	Sleep efficiency (%)	85.16 (5.14)	86.12 (5.51)	−0.69 (56)	.49

### Validating the Appearance-Based Intervention

When participants in the appearance group were asked to select what they looked like, they identified Image 6.81 on average (SD 3.32). A one-sample *t* test found no evidence that this differed from the actual baseline image (Image 6; *t*_26_=1.28, *P*=.21, 95% CI for the mean difference: −0.50 to 2.13); in other words, participants correctly identified their current appearances.

**Figure 4:**
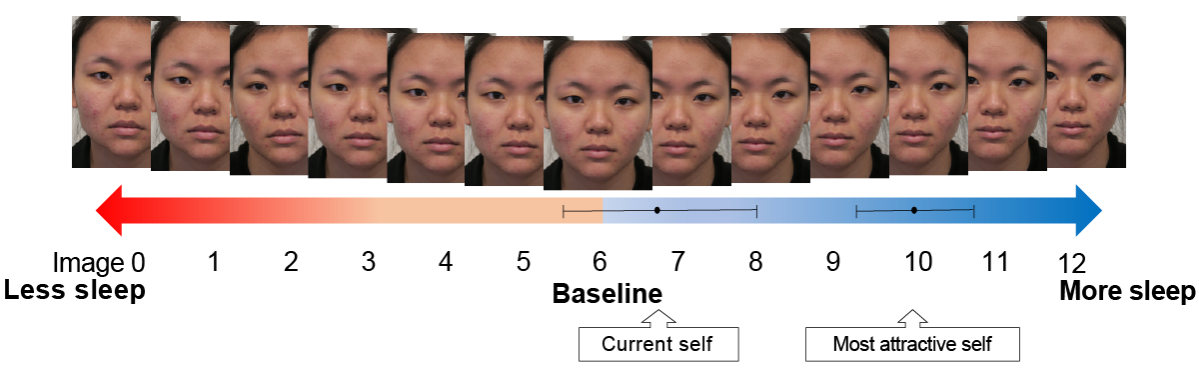
Participants viewed a continuum of 13 images transformed to show what they would look like with less or more sleep. When asked to identify their current selves, participants accurately identified an image close to the baseline (Image 6). They further judged that they would look more attractive following extended sleep. (Along the continuum, dots represent the mean images participants chose, and horizontal lines depict 95% CIs for the mean.).

On the other hand, participants selected Image 9.96 (SD 1.99) as the most attractive version of themselves. This differed from the baseline by an average increment of 3.96 steps (95% CI for the mean difference: 3.18-4.75; *t*_26_=10.35, *P*<.001; *d*=1.99). Together, the pattern of website clicks validates the appearance-based intervention, showing how participants (1) were sensitive to the photo transformations and (2) judged that they would look more attractive after a longer sleep duration (Figure 4).

### Effect of the Appearance-Based Intervention: Sleep Hygiene

**Figure 5:**
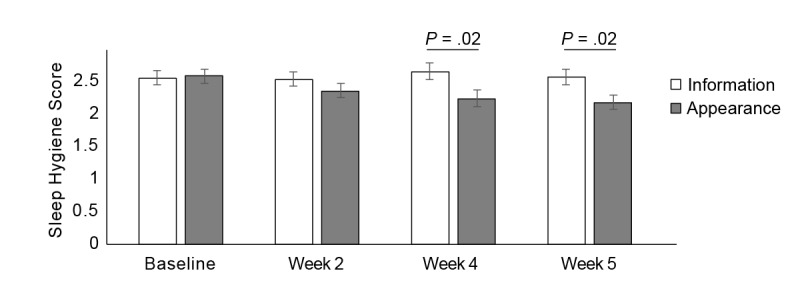
Mean sleep hygiene scores for participants in the information and appearance groups, plotted as a function of time (baseline and postintervention weeks 2, 4, and 5). A higher score corresponds to poorer sleep hygiene, and vertical lines represent 1 standard error of the mean.

Compared with a standard information program, the appearance-based approach was more successful in changing behaviors that could result in better sleep. Namely, on the sleep hygiene measure, participants in the appearance group showed improved sleep hygiene after the intervention (effect of time: *F*_1,107.99_=9.05, *P*=.003), whereas those in the information group did not show significant changes (effect of time: *F*_1,84.7_=0.19, *P*=.66; Figure 5). Comparing across time points, no group differences were observed at baseline or at week 2 postintervention (smallest *P*=.27). However, the appearance group reported better sleep hygiene than the information group in week 4 (*F*_1,38_=6.14, *P*=.02; 95% CI: −0.78 to −0.08; η_p_²=0.14) and week 5 (*F*_1,49_=6.23, *P*=.02; 95% CI: −0.68 to −0.07; η_p_²=0.11).

### Effect of the Appearance-Based Intervention: Actigraphy-Measured Sleep Duration

**Figure 6:**
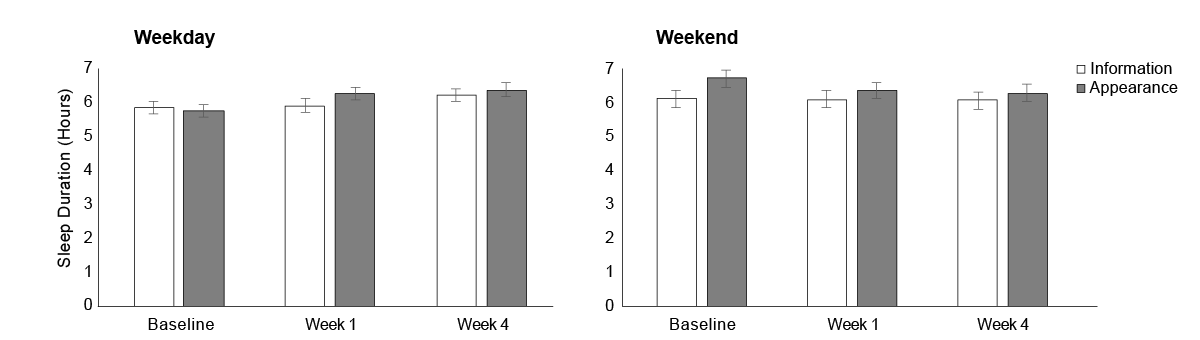
Mean sleep duration of participants in the information and appearance groups, plotted as a function of time (baseline and postintervention weeks 1 and 4). Vertical lines represent 1 standard error of the mean.

As shown in Figure 6, there was neither any significant effect of time on weekday sleep duration (*F*_2,96.71_=0.19, *P*=.83) nor a significant interaction between time and group (*F*_2,96.71_=1.38, *P*=.26). For weekend sleep duration, there was again no significant effect of time (*F*_2,103.77_=0.50), nor a time × group interaction (*F*_2,103.77_=0.16, *P*=.85; Figure 5). In Multimedia Appendix 2, Table S1 shows adjusted means for the primary mixed-effects analyses, and Multimedia Appendix 2, Table S2 shows the corresponding unadjusted means. Finally, Multimedia Appendix 2, Table S3 shows how sleep quality and insomnia-related cognition remained stable throughout the trial (smallest *P* for time or the time-group interaction=.48).

## Discussion

### Principal Findings

In this study, we developed a Web-based appearance-based intervention to promote sleep among habitual short sleepers. As a proof of concept, we first confirmed that participants (1) were sensitive to appearance changes and (2) judged their well-rested selves as more attractive. Correspondingly, this approach was more effective than a standard information program in promoting sleep hygiene, with benefits sustained for 1 month after the intervention.

Although these results are promising, the appearance-based intervention did not alter sleep duration itself. One possible explanation is that many paths lead to short sleep, of which a motivational component is only one. Thus, while appealing to attractiveness likely motivated change in presleep habits, it was not sufficient to alter sleep patterns. This account would suggest the need for a multipronged approach by public health agencies: for example, if sleep curtailment occurs because of immediate priorities such as pressing deadlines [16,17], an appearance-based intervention needs to be paired with techniques that help participants address their immediate priorities [44]. Alternatively, the intervention could be paired with structural changes such as a delayed school start time or reduced work hours, both of which have been found to increase sleep duration [45-47]. Further research will need to explore whether these pairings will ultimately address short sleep and how exactly motivation plays a role.

More broadly, our findings add to the growing body of research showing how an emphasis on appearances—though not sufficient as a stand-alone—may supplement traditional education in public health [25,26,28]. In recent years, appearance-based interventions have gained traction through the technological advances of photo-transformation software. These have been implemented in mobile- and Web-based applications [27,48,49] and have been applied across a range of health domains (sun protection [25], smoking cessation [26], nutrition [28], and—with this study—sleep). Given the potential reach of internet dissemination, one way forward may be to create a single appearance-based intervention that targets multiple health domains. If introduced in a timely manner (eg, incorporated into photo uploading features on a mobile dating application), this could boost existing public health campaigns, motivating healthy behaviors in a cost-effective manner.

### Study Limitations

In presenting our study, we note several limitations in participant selection and randomization. First, we targeted participants with habitual short sleep, departing from earlier sleep education studies where participants were recruited regardless of sleep history [11]. Accordingly, we could not assess how the appearance-based intervention would affect the broader population. Second, we recruited university students, a group whose schedules are driven by high-stake deadlines within a short 13-week semester. It remains possible that stronger treatment effects would be observed in groups without similar deadlines (eg, adolescents or young working adults). Third, in choosing university students, we sought—in a proof-of-concept trial—to maximize the potential impact of our intervention. Across adulthood, although the likelihood of being a short sleeper is fairly even across age groups [50], concerns about physical appearances peak in young adults (≤24 years) and decrease linearly across the life span [51]. We thus reasoned that the selected age group (18-24 years) would be most amenable to an appearance-based intervention. However, in choosing these age cutoffs, we did not assess directly whether participants valued their appearances and recognize that our findings may not generalize to other age groups—for example, to older participants whose appearances may be affected by normal aging. Moving forward, selection of the appearance-based strategy can thus be customized based on the importance of physical attractiveness to the individual. Finally, as an eHealth trial, we were unable to blind the identity of which intervention participants were randomized to.

### Conclusions

In conclusion, we designed in this study a novel intervention emphasizing physical attractiveness as a function of sleep patterns. This was assessed through a rigorous randomized controlled trial design with the following [52]: (1) the key intervention personalized (following best practices in appearance-based interventions [28]), (2) sleep duration objectively assessed (via actigraphy), and (3) participants monitored for 5 weeks. Using this design, we found that the appearance-based intervention was more effective than standard education in promoting sleep hygiene, a precursor for healthier sleep. At the same time, these effects did not translate to sleep extension, suggesting the need to assess how appearance-based strategies can be paired with other interventions. In summary, while urging further replication and extension of this work, our preliminary results suggest that beauty—the driving force that launched a thousand ships—may have an adjunctive role in promoting sleep.

## Appendix

Multimedia Appendix 1 Informed consent documentation.

Multimedia Appendix 2 Tables S1-3: Additional analyses.

Multimedia Appendix 3 CONSORT-eHEALTH checklist (V 1.6.1).

## Abbreviations

DBAS-16: Dysfunctional Beliefs and Attitudes about Sleep scale
PSQI: Pittsburgh Sleep Quality Index
