# Supplementary material for: A Web-Based Photo-Alteration Intervention to Promote Sleep: Randomized Controlled Trial
Source: J Med Internet Res. 2019 Sep 26;21(9):e12500. doi: 10.2196/12500 (PMC7017650; doi:10.2196/12500)
Supplement: Multimedia Appendix 1 [file jmir_v21i8e12500_app1.pdf]

AN EVALUATION OF INTERVENTIONS FOR THE PROMOTION OF SLEEP

Principal Investigator:
Dr. Jean Liu, Yale-NUS College, jeanliu@yale-nus.edu.sg

Co-Investigators:
Dr. David Perrett, University of St. Andrews, dp@st-andrews.ac.uk
Dr. Michael Chee, Duke-NUS Graduate Medical School, michael.chee@duke-nus.edu.sg

You are invited to participate in a research study. This information sheet provides you with information about the research. The Principal Investigator or her representative will also describe this research to you and answer all of your questions. Read the information below and ask questions about anything you don't understand before deciding whether or not to take part.

Purpose of research:

This research seeks to evaluate campaigns designed to promote sleep.

Who can participate:

This study will recruit 70 healthy NUS students between the ages of 18 and 24. Participants should not have any pre-existing medical, sleep or psychiatric conditions, nor a history of substance abuse.

Expected duration of participation:

Approximately 4-5 weeks.

If you participate, you will be asked to:

- Visit the lab <u>five (5) times</u> for the duration of the study
  - Visit 1: Signing of the consent form, briefing, completion of questionnaires, collection of Actiwatch and sleep diaries, photo-taking (30 minutes)
  - Visit 2 (1 week after Visit 1): Exposure to sleep campaign (2 hours)
  - Visit 3 (1 week after Visit 2): Return of Actiwatch and sleep diaries, completion of questionnaires and photo-taking (30 minutes)
  - Visit 4 (2 weeks after Visit 3): Collection of Actiwatch and sleep diaries, completion of questionnaires and photo-taking (30 minutes)
  - Visit 5: (1 week after Visit 4) Return of Actiwatch and sleep diaries, debriefing (30 minutes)
- Participate in a sleep promotion campaign. This could involve: reading brochures, completing computer tasks, and/or discussions with the researcher.
- As part of this study, your photograph will be taken.
- Wear an Actiwatch for 3-4 weeks.
  - You must take good care of your Actiwatch and wear them at all times except when bathing or swimming.
  - The Actiwatch is a watch-shaped product designed for research. Similar to a 'sports watch' that records how many steps a person takes during a day, the Actiwatch has motion sensors which record amount of activity as a marker of whether the user is sleeping. It also has light sensors to track light exposure.
  - The use of the Actiwatch is non-invasive and safe.
- Track and report sleep activity during the weeks the Actiwatch is used.
- You may be contacted and reminded to fill out your sleep diaries on a daily basis (once a day) via text/call/email.

At the end of the experiment, your pre- and post-intervention photographs will be compared. As part of this comparison, the photographs may be rated by members of the research team or by external parties (e.g., other university students).

How will my privacy and the confidentiality of my research records be protected?

Only the experimenters will have access to the names, contact numbers, and emails of the participants. All other data collected (including the photographs) will be assigned codes for

anonymity, and will not be released to any persons not involved in the study. Although your name is not associated with the photographs, you may still be identifiable. However your photographs will not be used for publications / presentations.

In line with the NUS Research Data Management Policy, all research data will be kept for a minimum of 10 years before being discarded. All data not used in publication (including personal identifiers) will be deleted at the end of the research study if you do not consent to its use in future related studies or to be re-contacted.

What are the possible discomforts and risks for participants?
None. There are no foreseeable discomforts, risks or potential for participant injury in this study.

Will there be reimbursement for participation?
As compensation for your time, you will be reimbursed $50 upon study completion.

What are the possible benefits to me and to others?
There is no direct benefit to you by participating in this research study. However your participation will further our understanding of sleep habits and of how to improve sleep promotion campaigns. You may also gain insights into your own sleeping patterns.

Can I refuse to participate in this research?
Your participation in this study is completely voluntary. You are entitled to refuse to participate or discontinue participation at any time in this research.

Should you decide to withdraw from the study, you may do so at any point without providing an explanation and without any negative consequences; you simply need to inform the researcher(s) and return the Actiwatch. If you decide to withdraw, any data collected from you will be discarded and will not be further used.

If you have any questions about this study, please contact:

- Principal Investigator: Dr Jean Liu, Asst. Professor, Yale-NUS College, 6 College Avenue East, Singapore 138614, jeanliu@yale-nus.edu.sg, +65 6601 2694

- For an independent opinion regarding the research and the rights of research participants, you may contact a staff member of the National University of Singapore Institutional Review Board (Attn: Mr Chan Tuck Wai, at telephone (+65) 6516 1234 or email at irb@nus.edu.sg).

Consent Form

Project title: An evaluation of interventions for the promotion of sleep
Principal Investigator: Dr Jean Liu, Yale-NUS College, jeanliu@yale-nus.edu.sg

I hereby acknowledge that:

1. My signature is my acknowledgement that I have agreed to take part in the above research.

2. I have received a copy of this information sheet that explains the use of my data in this research. I understand its contents and agree to donate my data for the use of this research.

3. I can withdraw from the research at any point of time by informing the Principal Investigator and all my data will be discarded.

4. I will not have any financial benefits that result from the commercial development of this research (if any).

5. I agree to my photo being taken (and possibly rated) as part of my participation in the research. I understand although my name is not associated with the photographs, I may still be identifiable. However these photographs will not be used in publication or presentations.

Additionally:

1. I consent / do not consent* to have my coded data (without personal identifiers) made available for future related research. I understand that future related studies will be subject to an Institutional Review Board's approval.

2. I agree / do not agree* to be re-contacted for participation in future related studies.

________________________________ ___________

Name and Signature (Participant) Date

________________________________ ___________

Name and Signature (Consent Taker) Date
