# Supplementary material for: A Web-Based Photo-Alteration Intervention to Promote Sleep: Randomized Controlled Trial
Source: J Med Internet Res. 2019 Sep 26;21(9):e12500. doi: 10.2196/12500 (PMC7017650; doi:10.2196/12500)
Supplement: Multimedia Appendix 2 [file jmir_v21i8e12500_app2.pdf]

Table S1. Intervention effects on actigraphy-measured sleep duration (adjusted means)

Outcome Measure[1]	Mean (95% confidence interval)					
	Baseline (Week 0)		Post-intervention (Week 1)		Post-intervention (Week 4)	
	Information	Appearance	Information	Appearance	Information	Appearance
Weekday sleep duration (hours:mins)	5:50 (5:29, 6:11)	5:46 (5:24, 6:07)	5:55 (5:32, 6:18)	6:17 (5:55, 6:39)	6:13 (5:51, 6:35)	6:23 (6:00, 6:45)
Weekend sleep duration (hours:mins)	6:06 (5:36, 6:06)	6:42 (6:12, 7:13)	6:05 (5:34, 6:36)	6:21 (5:52, 6:51)	6:04 (5:34, 6:34)	6:17 (5:47, 6:47)

[1]Adjusted for baseline scores on the Pittsburgh Sleep Quality Index (PSQI).

Table S2. Intervention effects on actigraphy-measured sleep duration (unadjusted means)

Outcome Measure	Mean (95% confidence interval)					
	Baseline (Week 0)		Post-intervention (Week 1)		Post-intervention (Week 4)	
	Information	Appearance	Information	Appearance	Information	Appearance
Weekday sleep duration (hours:mins)	5:55 (5:34, 6:16)	5:53 (5:32, 6:13)	5:58 (5:37, 6:19)	6:16 (5:56, 6:37)	6:19 (5:58, 6:40)	6:19 (5:58, 6:40)
Weekend sleep duration (hours:mins)	6:08 (5:39, 6:36)	6:41 (6:13, 7:09)	6:13 (5:45, 6:42)	6:23 (5:56, 6:50)	6:16 (5:48, 6:44)	6:22 (5:53, 6:51)

Table S3. Characterization of sleep quality and insomnia-related cognition across the trial

Outcome Measure	Mean (95% confidence interval)							
	Baseline (Week 0)		Post-intervention (Week 2)		Post-intervention (Week 4)		Post-intervention (Week 5)	
	Information	Appearance	Information	Appearance	Information	Appearance	Information	Appearance
Sleep quality[1]	10.63 (9.89, 11.38)	9.57 (8.84, 10.31)	10.61 (9.79, 11.42)	9.71 (8.90, 10.51)	10.62 (9.79, 11.46)	9.61 (8.72, 10.51)	10.12 (9.31,10.93)	9.27 (8.46, 10.08)
Insomnia-related cognition[2,3]	4.48 (3.96, 5.00)	4.63 (4.12, 5.14)	4.40 (3.84, 4.96)	4.41 (3.87, 4.95)	4.31 (3.71, 4.91)	4.53 (3.91, 5.16)	4.54 (3.97, 5.11)	4.36 (3.83, 4.89)

[1]As measured by global scores on the Pittsburgh Sleep Quality Index (PSQI).
[2]As measured by the Dysfunctional Beliefs and Attitudes about Sleep scale (DBAS-16).
[3]Adjusted for baseline PSQI.
